# Neuroprotective effect of Curcumin involved in increasing the protein levels of UCP2 and inhibiting oxidative stress induced by chronic cerebral ischemia in vitro

**DOI:** 10.1186/1750-1326-7-S1-S26

**Published:** 2012-02-07

**Authors:** Minna Gao, Li Liu, Peng Zhang, Gang Yu, Yu Li

**Affiliations:** 1 Department of Pathology, Yongchuan Hospital, Chongqing Medical University, China; 2 Institute of Neuroscience, Chongqing Medical University, China; 3 Chongqing Key Laboratory of Neurobiology, China; 4 Department of Neurology, The First Affiliated Hospital, Chongqing Medical University, Chongqing 400016, China

## Background

Chronic cerebral ischemia is caused by the long-term cerebral hypoperfusion, it is a common pathological process that usually occurs in conditions such as Alzheimer’s disease and vascular dementia. Oxidative stress plays an important role in nerve cell damage, UCP2 (The uncoupling protein 2) is one member of UCPs, which are a family of mitochondrial anion-carrier proteins and plays an important role in inhibiting oxidative stress. This study aims to observe the antioxidation of Curcumin on chronic cerebral ischemia established by PC12 ischemic cell models and investigate the changes of UCP2 during chronic ischemic PC12 cell.

## Method

The PC12 ischemic cells were cultured in medium contain sodium hyposulfite (the final concentration was 1M). Cells were maintained at 37^o^C in an incubator containing 5% CO_2_, and changed to normal medium after24h-48h, then treated with different concentrations of Curcumin ( 0, 1.25, 5.0, 20 μmol/L) for 24h. The fluorescent probe DCFH-DA and fluorescent spectrophotometer were performed to detect the levels of the active oxygen, to observe the protective effect of Curcumin on PC12 cells. The expressions of UCP2 protein were detected by immunohistochemistry.

## Results

In vitro the levels of the active oxygen decreased in chronic ischemic PC12 cells after treated with Curcumin, and the changes were in a dose-dependent manner (p<0.05). The expression of UCP2 protein significantly increased after treated with Curcumin, and the changes were also in a dose-dependent manner (p<0.05).

## Conclusion

Our data demonstrated the obvious neuroprotective effect of Curcumin involved in increasing the protein levels of UCP2 and inhibiting oxidative stress induced by chronic cerebral ischemia in vitro. Further studies are needed to firmly establish this protective effect.

